# Prospective Evaluation of the Correlation of Lung Ultrasonography Score and Blood Gas Parameters in Neonates With Respiratory Distress

**DOI:** 10.7759/cureus.41716

**Published:** 2023-07-11

**Authors:** Umit Ayse Tandircioglu, Nuriye Asli Melekoglu

**Affiliations:** 1 Department of Pediatrics, Kırıkkale University, Kırıkkale, TUR; 2 Department of Pediatrics, Malatya Turgut Özal University Faculty of Medicine, Malatya, TUR

**Keywords:** lung ultrasound, respiratory distress, newborn, blood gas sample, lung ultrasound score

## Abstract

Introduction

Lung ultrasonography (LUS) has become frequently used in neonatal intensive care units (NICU) because it is diagnostic, useful, harmless, radiation-free, and practical for bedside use due to its portability.

Objective

This study aimed to evaluate the association between lung ultrasound (LUS) scores and diagnoses of neonates hospitalized for respiratory distress and determine the value of the combined use of laboratory and imaging methods in patient evaluation by looking at the correlation between blood gas parameters and LUS score.

Materials and methods

Between March and July 2022, a total of 55 patients who were born term or premature and admitted due to respiratory distress in the NICU of Malatya Training and Research Hospital were included in the study. In this observational, prospective study, demographic information such as birth weights, gestational weeks, mode of delivery, Apgar scores, blood gas sample results, LUS results and scores, ventilation types, and discharge time were recorded during hospitalization in our unit.

According to the newborns' clinical, laboratory, and radiologic evaluations, the diagnoses of respiratory distress syndrome (RDS), transient tachypnea of the newborn (TTN), or congenital pneumonia were made, and the relationship between the diagnoses and LUS scores was evaluated. The pH value and PCO2 value in the venous blood gas obtained on the day of LUS were recorded. Correlation analysis was performed between the LUS score and pH value, LUS score and PCO2 value.

Results

Twenty-seven newborns were diagnosed with TTN, 18 with RDS, and 10 with congenital pneumonia. There was a statistical difference between LUS scores and diagnoses (p<0.001). According to Spearman correlation analysis, a significant negative moderate correlation was found between LUS scores and venous blood gas pH value (p<0.001, r:-0.49). There was also a significant positive low, moderate correlation with venous blood gas PCO2 value (p<0.001, r:0.36).

Conclusion

This study demonstrates that LUS scoring has a role in determining the severity of disease and making diagnoses in patients hospitalized for respiratory distress. When LUS is widely used, it will be informative about the severity and prognosis of the disease, together with laboratory evaluation.

## Introduction

Lung ultrasonography (LUS) was first introduced in adult care in 1995, and in recent years, its use has expanded into neonatology and pediatrics, becoming increasingly widespread. Due to its diagnostic, useful, harmless, radiation-free, bedside, reproducible, and practical attributes, lung ultrasonography has become a frequently used method in neonatal intensive care units (NICUs) [1, 2]. In our clinic, all infants hospitalized with respiratory distress are imaged with LUS in the first stage. LUS is widely used in conditions that mainly cause respiratory distress, such as respiratory distress syndrome (RDS), pneumonia, transient tachypnea of the newborn (TTN), congenital pneumonia, meconium aspiration syndrome (MAS), and pneumothorax [1, 2]. The reliability and specificity of LUS imaging, especially in diagnosing RDS, have been proven in many studies [1, 2,3]. The diagnosis is made by visualizing the 'transition zone (double lung point)' on LUS [3]. 'White lung appearance' in RDS, 'absence of pleural motion' and 'barcode appearance' in M mode in the presence of pneumothorax (one of the air leakage syndromes), and 'hepatization/consolidation appearance' in pneumonia are among the ultrasonographic findings [1, 3]. In recent years, the first diagnostic imaging modality for infants with respiratory distress in the NICU was the chest X-ray. Today, however, LUS has taken priority. In this way, radiation exposure of newborns is reduced. In our unit, LUS is performed primarily in infants with respiratory distress, and a chest X-ray is not performed unless respiratory distress persists. In early or late preterm infants whose LUS findings are compatible with RDS and who clinically require surfactant, surfactant treatment is given in a shorter time without waiting for a chest X-ray. In a study involving 40 patients diagnosed with RDS, there was a significant improvement in LUS in the 4th hour of surfactant treatment [4]. In a previous study, when a study group of 104 newborns was compared with a historical control group of 73 newborns, it was found that the widespread use of lung ultrasonography reduced the need for chest X-rays in patients admitted to the NICU for respiratory distress. [5]. LUS reduces chest X-ray exposure and protects against the effects of radiation. In addition, fast and practical results were obtained due to bedside ultrasound. LUS monitoring has now become an objective value by scoring. Efforts have been made to determine the relationship between these scores and the diagnosis of the disease [6,7]. Our study aimed to evaluate the relationship between the LUS score and the diagnosis of neonates hospitalized because of respiratory distress. Venous blood gas measurement is one of the parameters used to evaluate respiratory distress in newborns. In this evaluation, partial carbon dioxide and pH measurements indicate the respiratory status of the newborn. Many patients with respiratory distress are diagnosed early by using LUS in newborns. Scoring using LUS is valuable because it provides an unbiased result. It is also known that blood gas values give an idea about the severity of the disease in newborns with respiratory distress. In our study, we aimed to evaluate the correlation between LUS scores and blood gas parameters in patients hospitalized for respiratory distress and to get an idea about the severity of the disease.

## Materials and methods

**Figure 1:**
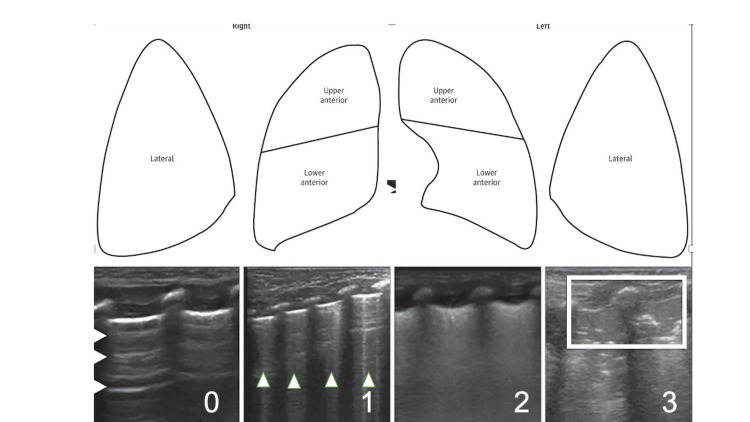
Description of the LUS score. Each lung has been divided into three areas, as shown in the upper part of the figure. For each area, a score of 0 to 3 has been assigned. Scores are given as follows, for any lung area: 0 indicates only A lines ( arrowheads); 1, defined as the presence of ≥3 B-lines (arrowheads); 2, severe B lines without consolidations (white lung) and 3, extended consolidation (box). LUS: Lung ultrasound.

After ethics committee approval (decision no: 2022/155) was obtained, 55 patients who were born term or premature and admitted due to respiratory distress between March and July 2022 in the NICU of Malatya Training and Research Hospital were included in the study. In this observational, prospective study, demographic information such as birth weights, birth weeks, mode of delivery, Apgar scores, blood gas sample results, LUS results and scores, ventilation types, and discharge time during hospitalization in our unit were recorded. Imaging was obtained using the linear probe of an ultrasound device (Esaote MyLab Seven) and LUS scoring as described in the studies of Brat R et al. and Raimondi F et al. [8,9]. Accordingly, both lungs were evaluated as three areas on the right and left (anterior superior, anterior inferior, and lateral) and scored separately. The region between the anterior axillary and parasternal lines was divided into two, with a line passing through the nipple. The upper region was evaluated as upper anterior, and the lower region as lower anterior (Figure 1). The area in the middle of the anterior and posterior axillary line was evaluated as the lateral region. Each area is scored from 0 to 3 points. If A lines are present, 0 points are given; if there are more than three B lines in an area, 1 point is given; if B lines are very dense and A lines are absent, it is considered white lung and 2 points are given; if there is a consolidation appearance on LUS, 3 points were given (Table 1).

**Table 1:** Definiton of LUS scores. LUS: Lung ultrasound.

LUS Score	Definition
0	‘A’ lines are present
1	More than 3 ‘B’ lines in an area
2	B lines are very dense and A lines are absent (white lung)
3	Consolidation appearance

The total score was evaluated as a maximum of 18 [8,9]. According to the clinical, laboratory, and radiologic evaluations of the patients, the diagnoses of RDS, TTN, or congenital pneumonia were made, and the relationship between the diagnoses and LUS scores was evaluated. The pH value and PCO2 value in the venous blood gas obtained on the day of LUS were recorded. Correlation analysis was performed between the LUS score and pH value, and LUS score and PCO2 value. SPSS version 28 (SPSS, Chicago, IL, USA) was used for statistical analysis. Data were expressed as mean ± SD, median (minimum-maximum value), percentage, and ratio. The variables were analyzed for normal distribution using the Shapiro-Wilk test. Mean and SD values were written for the data that matched the normal distribution, and median and minimum-maximum values were written for the data that did not match the normal distribution. Since LUS scores and diagnoses were non-parametric variables, the Kruskall-Wallis test was used, and a p-value of <0.05 was determined as the significance limit. Spearman test was used for the correlation analysis of non-parametric data, the student's t-test was used for comparing parametric variables, and Pearson correlation analysis was used for the correlation analysis of parametric variables.

## Results

Among the patients admitted to our clinic due to respiratory distress, 27 were diagnosed with TTN, 18 with RDS, and 10 with congenital pneumonia. None of the patients had MAS or pneumothorax. The clinical, laboratory, and demographic characteristics of these patients are shown in Table 2.

**Table 2:** Clinical and demographic characteristics. * median (min-max) TTN: Transient tachypnea of the newborn; RDS: Respiratory distress syndrome.

Demographic datas		TTN (n=27)	RDS (n=18)	Congenital pneumonia (n= 10)	P-value
Birth weight (g)*		3200 (1770-4200)	1170 (680- 1890)	2870 (2285-3600)	<0.001
Gestational age*		38 (32-41)	28 (26-31)	37 (34-39)	<0.001
Gender(male)		22 (82%)	10 (56%)	4 (40%)	0.37
APGAR (1./5. Dk) *		8(7-9) / 9(8-9)	6(4-7) / 7(5-8)	8(7-9) / 9(8-10)	<0.001/ <0.001
Type of delivery (C/S)		17 (63%)	15 (83%)	7 (70%)	0.343
Length of stay*		2 (1-7)	44 (32-103)	8 (7-31)	<0.001
Ventilation type on the postnatal first day	Invasive ventilation	0	13 (72%)	2 (20%)	<0.001
	Non-invasive ventilation	19 (70%)	5 (28%)	6 (60%)	
	Only oxygen	8 (30%)	0	2 (20%)	
Venous blood gas pH value *		7.33 (7.17-7.40)	7.24 (7.02-7.33)	7.3 (7.2-7.4)	0.016
Venous blood gas pCO2 value (mmHg)*		42.7 (35-75)	53 (37-83)	46 (28-66)	<0.001

LUS imaging times and scores of the patients according to their diagnoses are shown in Table 2. A statistical difference was found in LUS scores across different diagnoses (p<0.001). LUS imaging time, diagnoses, and scores are shown in Table 3.

**Table 3:** LUS imaging time and scores. *median (min-max) † median (min-max), p<0.001 TTN: Transient tachypnea of the newborn; RDS: Respiratory distress syndrome; LUS: Lung ultrasound.

Diagnosis of newborns	Lung ultrasound screening time (hour)*	LUS †
TTN	8 (1-15)	3 (1-8)
RDS	3 (2-16)	12 (3-18)
Congenital pneumonia	15 (1-24)	8 (3-12)

**Figure 2:**
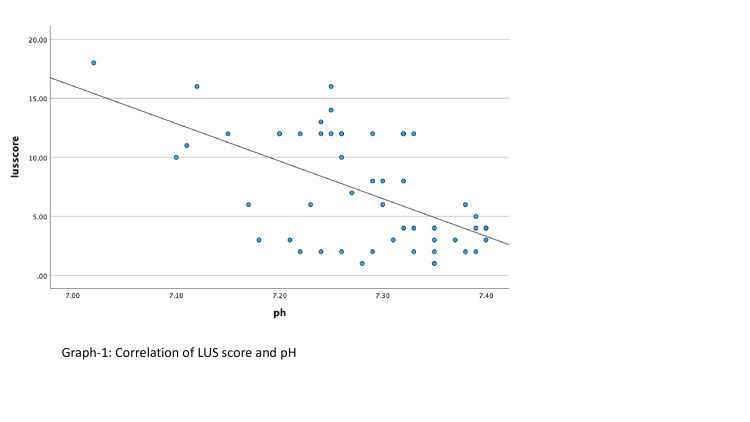
Correlation of LUS score and pH. LUS: Lung ultrasound.

**Figure 3:**
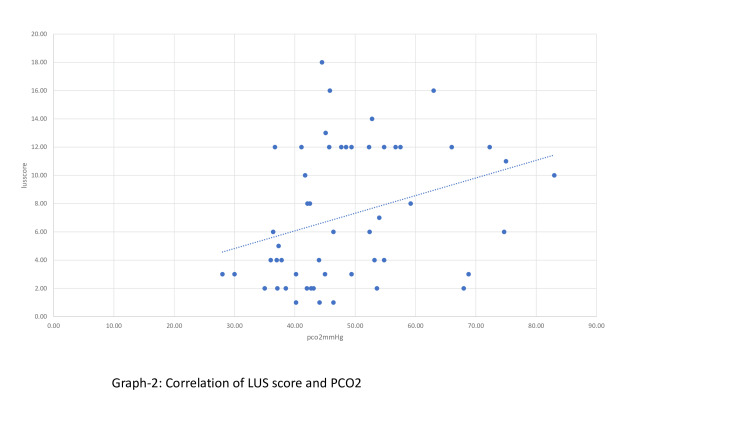
Correlation of LUS score and PCO2. LUS: Lung ultrasound.

According to the Spearman correlation analysis, a significant negative moderate correlation was found between LUS scores and venous blood gas pH value (p<0.001,r:-0.49) (Figure 2). A significant positive low, moderate correlation (p<0.001, r:0.36) was found with venous blood gas PCO2 value (Figure 3).

## Discussion

Our study evaluated the relationship between LUS score, diagnoses, and the correlation between blood gas parameters. Currently, LUS is widely used in NICUs. It has a sensitivity of 96.7% and a specificity of 100% in diagnosing RDS and an agreement rate of 96.7% with chest X-ray, which is the gold standard in diagnosing RDS [10,11]. It has a sensitivity of 100% and a specificity of 97.8% in the diagnosis of TTN and an agreement rate of 98.4% with chest X-ray. When TTN is diagnosed with LUS in newborns with respiratory distress, unnecessary antibiotic use will be prevented, and a calmer and gentler follow-up will be realized. Although there are not many studies on congenital pneumonia, the diagnosis can be supported by LUS findings [11]. Especially in newborns diagnosed with RDS, there is much data on the importance of LUS score in determining the surfactant requirement. In the study by Brat R et al., LUS score performed within a few hours postnatally was correlated with oxygen indices and predicted the need for surfactant [8]. Studies have shown that LUS can predict the need for surfactants more accurately than the fraction of inspired oxygen (FiO2) [12,13]. With the use of LUS, surfactant therapy can be administered to infants diagnosed with RDS in the early period before their FiO2 needs increase too much and lung injury develops, and thus RDS complications occur. In addition, the use of chest X-rays can be reduced with the use of LUS in the follow-up after surfactant therapy [4,5]. The use of LUS also provides insight into the disease's prognosis and whether a re-surfactant will be needed. In 2021, Raimondi F et al. published a multicenter study showing that LUS score predicts the need for surfactant, and earlier surfactant administration, without waiting for an increase in FiO2 requirement for surfactant administration, also reduces oxygen requirement at follow-up [14]. The Echography-guided Surfactant THERapy (ESTHER) study used LUS score to guide surfactant administration, leading to earlier surfactant administration and a shorter duration of invasive ventilation without increasing cost [15]. Before this study, although studies were mostly related to surfactant administration, the relationship between LUS score and diagnoses was also evaluated, which we thought would contribute to the literature. We have shown that LUS score is valuable in differential diagnosis evaluation.

The study by Xi G et al. examined the correlation between the need for respiratory support and LUS in term and late preterm infants and observed that the need for respiratory support was higher in high-risk newborns [16]. In our study, we found a statistical difference between the severity of the diseases and LUS score. While the LUS score of newborns with RDS was the highest, the scores of newborns diagnosed with TTN were the lowest. This suggests that the LUS score may be a tool to determine the difference between diagnoses of respiratory distress in the newborn and evaluate disease severity. Similarly, in a study conducted by Pang H et al. in 2019, a significant difference was found when LUS scores of 96 patients with RDS and 50 patients with TTN were evaluated, and this difference was not observed in patients with mild RDS [7]. In our study, the fact that patients with RDS were not divided into mild and severe can be considered a limitation. Another study with 157 patients found that LUS scores were associated with neonatal respiratory distress, which suggested that the LUS score may provide information about the prognosis of neonatal respiratory distress [17]. In 1970, Downes JJ et al. developed a scoring system determining respiratory distress in infants with RDS and correlating it with blood gas parameters [18]. In this study, when we evaluated the correlation of LUS scoring with pH and pCO2 values among the blood gas parameters obtained, we found a significant negative moderate correlation between LUS scores and venous blood gas pH value. It was determined that as LUS scores increased, the pH value decreased as the severity of the disease increased. There was a significant positive low, moderate correlation between the LUS score and the PCO2 value of venous blood gas, indicating that the increase in LUS score was correlated with the increase in the PCO2 value of the newborn. Our study is very valuable since there is no study in the literature in which blood gas evaluation and LUS were evaluated together. There are several limitations to this study. The most important is that oxygenation could not be evaluated because venous blood gas measurement was performed. Also, the ratio of PAO2/FIO2 for oxygenation, emphasized in many studies, could not be compared. We have no data about the oxygenation index, so we cannot correlate it with the LUS score. Patients with RDS were not divided into mild or severe RDS, and evaluation was not performed. Unfortunately, the study did not record clinical evaluations of the patients (such as tachypnea, groaning). Another limitation is the small number of patients since the duration of the study was kept short. Future studies are planned to be conducted by increasing the number of patients. The last limitation is the diagnosis of pneumothorax. We use LUS to diagnose pneumothorax, but this study's timeline was narrow, so pneumothorax was not encountered. Because of this, we could not add patients with pneumothorax.

## Conclusions

This study demonstrates that LUS and LUS scoring, which are used more frequently in newborns and can be performed easily at the bedside without radiation exposure, have a role in determining the severity of the disease and diagnosing patients hospitalized for respiratory distress. When LUS is widely used, it will provide insights about the severity and prognosis of the disease, together with laboratory evaluation. Also, LUS scoring has the potential to aid in the diagnosis and management of neonates with respiratory distress, allowing for less need for chest X-rays and leading to earlier appropriate treatment.
